# Treatment with rGDF11 does not improve the dystrophic muscle pathology of *mdx* mice

**DOI:** 10.1186/s13395-016-0092-8

**Published:** 2016-06-14

**Authors:** Fabrizio Rinaldi, Yu Zhang, Ricardo Mondragon-Gonzalez, Jeffrey Harvey, Rita C. R. Perlingeiro

**Affiliations:** Lillehei Heart Institute, Department of Medicine, University of Minnesota, 4-128 CCRB, 2231 6th St. SE, Minneapolis, MN 55455 USA; Department of Neurology, The First Affiliated Hospital, Sun Yat-Sen University, Guangzhou, China; Departamento de Genética y Biología Molecular, Centro de Investigación y de Estudios Avanzados del IPN (CINVESTAV-IPN), México, D.F. Mexico; Tonus Therapeutics, Williamsville, NY USA

**Keywords:** GDF11, Muscle regeneration, Duchenne muscular dystrophy, Fibrosis

## Abstract

**Background:**

Duchenne muscular dystrophy (DMD) is an inherited lethal muscle wasting disease characterized by cycles of degeneration and regeneration, with no effective therapy. Growth differentiation factor 11 (GDF11), a member of the TGF-β superfamily and myostatin homologous, has been reported to have the capacity to reverse age-related skeletal muscle loss. These initial findings led us to investigate the ability of GDF11 to promote regeneration in the context of muscular dystrophy and determine whether it could be a candidate to slow down or reverse the disease progression in DMD.

**Results:**

Here, we delivered recombinant GDF11 (rGDF11) to dystrophin-deficient mice using the intra-peritoneal route for 30 days and evaluated histology and function in both steady-state and cardiotoxin-injured muscles. Our data confirmed that treatment with rGDF11 resulted in elevated levels of this factor in the circulation. However, this had no effect on muscle contractility nor on muscle histology. Moreover, no difference was found in the number of regenerating myofibers displaying centrally located nuclei. On the other hand, we did observe increased collagen content, which denotes fibrosis, in the muscles of rGDF11-treated dystrophic mice.

**Conclusions:**

Taken together, our findings indicate no beneficial effect of treating dystrophic mice with rGDF11 and raise caution to a potential harmful effect, as shown by the pro-fibrotic outcome.

**Electronic supplementary material:**

The online version of this article (doi:10.1186/s13395-016-0092-8) contains supplementary material, which is available to authorized users.

## Background

Duchenne muscular dystrophy (DMD), the most common muscular dystrophy in the young, is characterized by progressive muscle wasting, in which skeletal muscle fibers are replaced by nonfunctional fibrotic and connective tissue [1]. DMD is caused by mutations in the X-linked dystrophin gene, which is an essential element for the maintenance of muscle fiber integrity [2]. Dystrophin and its associated proteins play an important role in protecting myofibers from contraction-induced damage. Loss of dystrophin leads to destabilization of the extracellular membrane resulting in rapid and continuous damage of the structural integrity of myofibers [3]. Boys with DMD usually exhibit progressive motor difficulties and muscle wasting, and as a result, patients are wheelchair-bound by their teens, with eventual death due to cardiorespiratory insufficiency [4]. Current therapeutic methods are only palliative as to date no effective treatment is available for DMD, or any type of muscular dystrophy.

Growth differentiation factor 11 (GDF11), a member of the transforming growth factor β (TGF-β) superfamily, has been recently described to promote muscle regeneration in the context of aging [5]. GDF11 is highly homologous to myostatin, another TGF-β member also known as GDF8, sharing 89 % sequence identity throughout the mature active region. Accordingly, GDF11 and myostatin share the same receptors and similar signaling pathways [6, 7]. Despite this likeness, the aforementioned study suggested opposite effects to these ligands on skeletal muscle regeneration. Whereas myostatin has a well-known inhibitory effect on muscle growth and differentiation [8–11], this study demonstrated that reduced circulating levels of GDF11 are linked to age-associated muscle loss, also known as sarcopenia, and that increasing in its levels in old mice, upon a 30-day treatment with GDF11, reversed age-related dysfunction of the mouse skeletal muscle [5]. A similar anti-aging effect for GDF11 has also been reported for the cardiac muscle [12] and brain [13], where this growth factor has been demonstrated to reverse age-related cardiac hypertrophy and neurologic defects, respectively.

We tested here whether GDF11 could ameliorate the progression of the muscle wasting phenotype observed in DMD by promoting muscle regeneration. For this, rGDF11 was administered systemically to dystrophin-deficient *mdx* mice for 30 days. To further test the regenerative ability of GDF11-treated mice, we damaged muscles from a cohort of treated and control *mdx* mice with cardiotoxin (CTX). Our findings show that increased levels of GDF11 in dystrophic mice have no effect on skeletal muscle regeneration and function. In fact, we observed evidence of fibrosis in GDF11-treated mice, as shown by the higher levels of collagen deposition in the tibialis anterior muscle, raising caution to the use of GDF11 in DMD.

## Methods

### Mice

All animal studies were performed according to protocols approved by the University of Minnesota Institutional Animal Care and Use Committee. Five-week-old male *mdx* mice (C57BL/10ScSn-Dmdmdx/J; stock number 001801), purchased from Jackson Laboratories (Bar Harbor, ME, http://www.jax.org), were treated with GDF11 or vehicle, as detailed below. In a cohort of these mice, we promoted muscle injury by injecting 15 μl of CTX (10 μM, Sigma) into the tibialis anterior (TA) muscle.

### rGDF11 dosing and injections

We used the protocol used in the previous report for sarcopenia [5]. rGDF11 stock solution was prepared by dissolving it in water containing 0.1 % BSA and kept at pH 3.8 with HCL, according to manufacturer’s instructions. Dystrophic mice were given a daily single intraperitoneal (IP) injection of either rGDF11 (PeproTech, Inc.) at 0.1 mg/kg or vehicle (water containing 0.1% BSA pH 3.8; at the same volume as GFD11) for 30 days. After 30 days, most mice were analyzed. At this point, a cohort of treated mice was muscle damaged by receiving 15 μl of CTX (10 μM, Sigma) into the TA. Muscle regeneration was evaluated 10 days later. During the 10-day period, this cohort of *mdx* mice continued to receive daily IP injections of rGDF11 or vehicle.

### Muscle histology

For histology, the TA and diaphragm muscles were harvested, embedded in OCT compound (Leica Biosystems), and then frozen in isopentane cooled in liquid nitrogen. Serial 10 μm cryosections were collected. For quantification of myofiber size and numbers, sections were stained for laminin. Muscle sections were fixed in 4 % PFA for 30 min, washed three times with PBS, blocked with 5 % BSA in PBS for 1 h at room temperature (RT), and then incubated with rabbit anti-laminin antibody (1:400; Sigma) overnight at 4 °C overnight. The following day, slides were washed three times in PBS and then stained with goat Alexa-555 anti-rabbit secondary antibody (Invitrogen) for 45 min at RT. To stain nuclei, we used ProLong^®^ Gold Antifade reagent along with DAPI (molecular probes). Hematoxylin and eosin (H&E) staining was used for assessment of centrally located nuclei myofibers. Masson’s trichrome staining was utilized to detect collagen content (Polysciences, Inc.). Fiji ImageJ software was used to quantify myofiber size and numbers, as well as muscle cross-sectional area. To quantify the collagen, the colors from the figure were split and a threshold was used to identify the blue staining. Then, the complete muscle section was selected as a region of interest, and the percentage of area depicted by the threshold (equivalent to the collagen area) was measured also using ImageJ software.

### Muscle preparation for mechanical studies

For the measurement of contractile properties, mice were anesthetized with ketamine/xylazine (80 mg/kg) and the intact TA muscles were dissected and placed in an experimental organ bath filled with mammalian Ringer solution containing (mM): NaCl 120.5; NaHCO3 20.4; glucose 10; KCl 4.8; CaCl2 1.6; MgSO4 1.2; NaH2PO4 1.2; pyruvate 1.0, adjusted to pH 7.4. The chamber was perfused continuously with 95 % O2–5 % CO2 and maintained at a temperature of 25 °C. The muscles were stimulated by an electric field generated between two platinum electrodes placed longitudinally on either side of the muscle (square wave pulses 25 V, 0.2 ms in duration, 150 Hz). The muscles were adjusted to the optimum length (Lo) for the development of isometric twitch force, and a 5-min recovery period was allowed between stimulations. Optimal muscle length (Lo) and stimulation voltage (25 V) were determined from micromanipulation of muscle length and a series of twitch contractions that produced maximum isometric twitch force. For measuring fatigue time, muscles were stimulated for 1 min and the time for force to decline to 30 % of Fo was measured. In brief, after determination of Lo and measurement of maximum isometric tetanic force, total muscle cross-sectional area (CSA) was calculated by dividing muscle mass (mg) by the product of muscle length (mm) and 1.06 mg/mm^3^, the density of mammalian skeletal muscle. Specific force (sFo) was determined by normalizing maximum isometric tetanic force (F0) to CSA. All of the *mdx* mice used for muscle physiology were also analyzed for histopathology.

### Western blot analysis

Plasma samples were collected from rGDF11 and vehicle-treated *mdx* mice, as previously described [14], and quantified with the Bradford regent (Sigma). Briefly, samples were diluted in Laemmli buffer and boiled for 10 min at 100 °C. Equal amounts (75 μg) of protein from each single mouse were loaded onto 4–20 % gradient mini-PROTEAN^®^ TGX Stain-Free™ gels (BioRad Labs). Proteins were transferred onto polyvinyl difluoride (PVDF) membranes (Millipore), which were then blocked in BSA 5 % Tris-buffered saline (TBS) with 0.05 % Tween-20 (TBST) for 1 h at RT. Membranes were incubated with rabbit anti-GDF11 antibody (1:1000, Abcam) overnight at 4 °C. The following day, these were incubated with horseradish peroxidase (HRP)-conjugated secondary antibody (GE healthcare). Proteins were detected by Pierce enhanced chemiluminescence (ECL) substrate (Thermo Scientific).

### Statistics analysis

One-way or two-way analysis of variance (ANOVA) was used to analyze differences. When significant (*p* < 0.05), this was followed by the Tukey post hoc analysis.

## Results and discussion

### rGDF11 treatment has no effect on the muscle regeneration of *mdx* mice

**Fig. 1:**
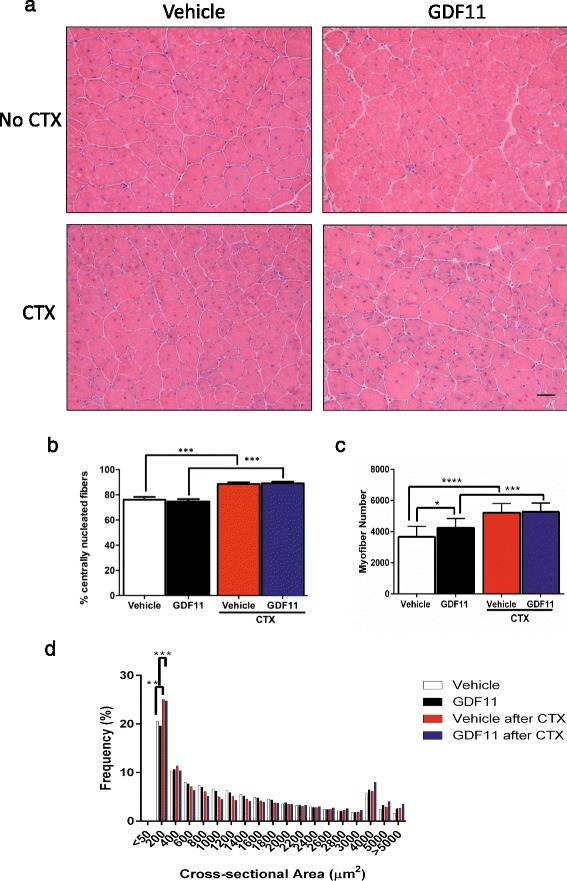
Histological analysis of the TA muscles isolated from *mdx* mice that had been treated with rGDF11 or vehicle. **a** Representative images of the TA muscles stained with H&E from mice treated with rGDF11 or vehicle (*upper panels*). Another cohort of mice treated with rGDF11 or vehicle was injured with cardiotoxin at day 30 and analyzed 10 days later (*lower panels*). **b** The percentage of centrally nucleated fibers was not altered upon rGDF11 treatment in mice with or without cardiotoxin injury. As expected, the cardiotoxin injury increased the percentage of centrally nucleated fibers. Treatment with GDF11 resulted in increased numbers of myofibers when compared to vehicle-treated mice (**c**) but did not affect the cross-sectional area frequency distribution (**d**). Quantification was performed in the entire cross sections of the tibialis anterior muscles. Both rGDF11- and vehicle-treated mice show leftward shift in frequency distribution. ≤200 μm^2^ myofibers were the largest group, and CTX injury increased the frequency of this group, irrespective of GDF11. Fiji ImageJ software was used to determine number and size of myofibers. *Scale bar* = 100 μm. *p < 0.05, **p < 0.01, ***p < 0.001, ****p < 0.0001

**Fig. 2:**
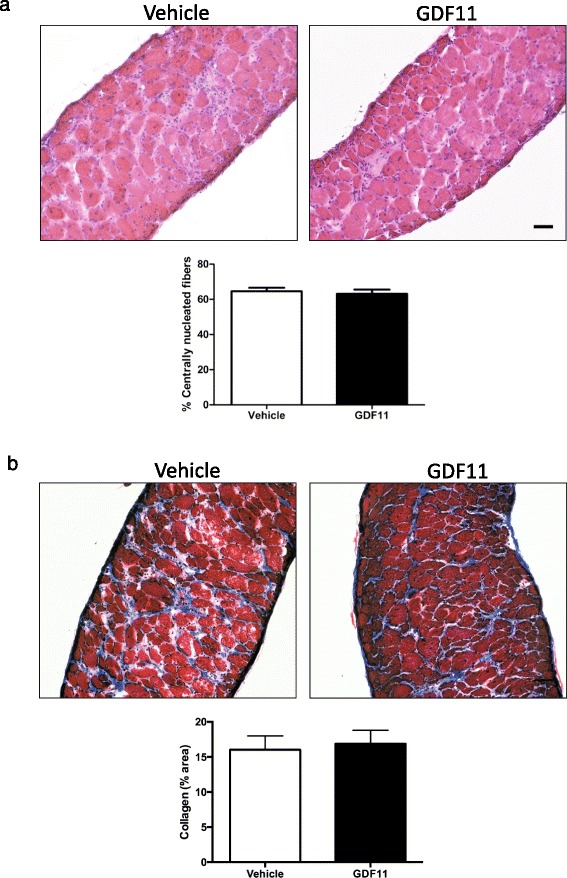
Histological analysis of the diaphragm muscle isolated from *mdx* treated with rGDF11 or vehicle. **a** Representative images of the diaphragm muscle using H&E staining from mice treated with rGDF11 or vehicle. The treatment with GDF11 did not alter the percentage of centrally nucleated fibers. **b** Representative images of the diaphragm muscle using Masson’s trichrome staining from mice treated with rGDF11 or vehicle. No differences were observed in terms of the percentage of fibrotic tissue upon rGDF11 treatment as determined by the quantification of the blue staining area (**b**). Fiji ImageJ software was used to quantify the percentage of collagen area. *Scale bar* = 100 μm

To determine the effect of GDF11 treatment on muscle regeneration in the context of muscular dystrophy, we treated *mdx* mice for at least 30 days with GDF11 (*n* = 16) or vehicle (*n* = 13). As expected, the higher levels of GDF11 were detected in the plasma of rGDF11-treated mice when compared to controls, as shown by western blot analysis (Additional file 1). For this, we used the GDF11 antibody from Abcam, which as previously reported [15] recognizes both the mature dimer (~25 kDa) and the monomer (~12.5 kDa) of both recombinant GDF11 and myostatin. We found increased levels of both, the monomer and the dimer, in the plasma of rGDF11-treated mice (Additional file 1). Nevertheless, by the end of the 30-day treatment, morphological analysis of the TA muscles by H&E staining showed no differences between GDF11- and vehicle-treated groups in regard to the number of regenerating centrally nucleated myofibers in the TA (Fig. 1a, upper panel, and b) and diaphragm (Fig. 2a) muscles. This was also the case for the cohort in which the TA muscles had been injured with CTX (Fig. 1a, lower panel, and b) following the 30-day treatment (rGDF11 or vehicle, *n* = 5 and *n* = 4, respectively), which was analyzed 10 days later, while receiving continuous administration of rGDF11 or vehicle. Although we observed the higher numbers of myofibers in the rGDF11-treated group in the absence of CTX injury (Fig. 1c, left), there was no difference between these groups upon injury (Fig. 1c, right). Both GDF11- and vehicle-treated muscles displayed equivalent increase in myofiber numbers following CTX injury (Fig. 1c), which consisted of small caliber regenerating fibers (Fig. 1d).

### Increased rGDF11 levels have no effect on muscle contractility

**Fig. 3:**
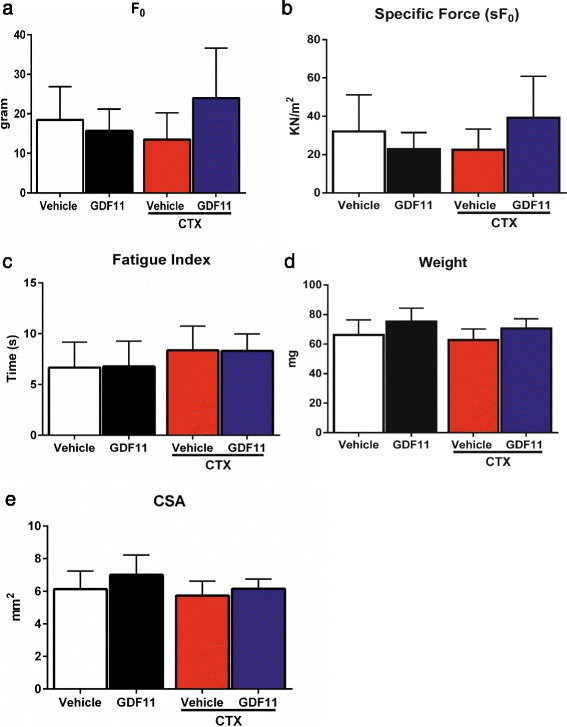
Assessment of muscle function. Isometric tetanic contraction was assessed in the isolated TA muscles from GDF11- and vehicle-injected *mdx* mice, in the presence or absence of CTX injury. No significant changes were observed in absolute force (F0) (**a**), specific force (sF0) (**b**), and fatigue index (**c**). No changes were observed in muscle weight or CSA (**d**, **e**, respectively)

To assess whether increased rGDF11 levels would improve the strength of dystrophic muscles, we measured the contractile properties of the TA muscles from rGDF11- and vehicle-treated *mdx* mice. No significant differences were observed in absolute or specific force (Fig. 3a, b), fatigue index (Fig. 3c), muscle weight, or CSA (Fig. 3d, e) between groups, both in the absence and in the presence of cardiotoxin injury.

### Higher collagen content in rGDF11-treated mice

**Fig. 4:**
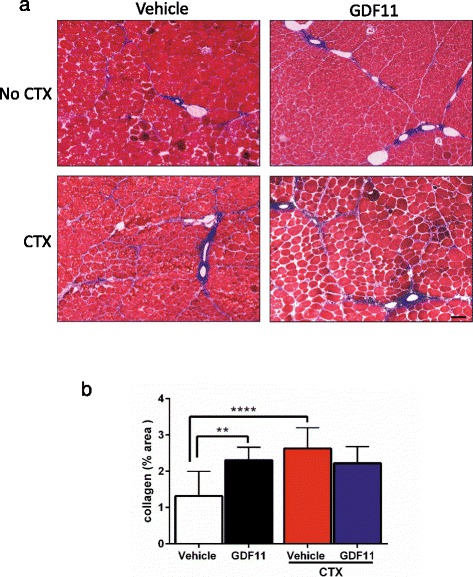
Assessment of fibrosis upon GDF11 treatment. **a** Representative images of the TA muscles stained with Masson’s trichrome. The presence of *blue* between myofibers represents collagen content, which is indicative of fibrosis. **b** Quantification of fibrosis shows GDF11-treated mice present increased levels of muscle collagen content in comparison with vehicle-treated mice. As expected, collagen content increased upon CTX injury, however, only in vehicle-treated mice. *Scale bar* = 100 μm. **p < 0.01, ****p < 0.0001

To determine levels of fibrosis, we evaluated collagen content in rGDF11- and vehicle-treated dystrophic muscles by Masson’s trichrome staining. Surprisingly, collagen content was significantly higher in the rGDF11-treated group when compared to that in the vehicle control (Fig. 4), which was not found to further increase upon CTX injury. On the other hand, injury in the vehicle group resulted in enhanced collagen content (Fig. 4b). No significant differences were observed between rGDF11 and vehicle groups in the presence of CTX (Fig. 4b) or in the diaphragm (Fig. 2b), suggesting that maximal levels of fibrosis had been already reached for the GDF11 group.

The role of GDF11 in skeletal muscle regeneration has been controversial [8, 12, 15–18]. While the previous report by Sinha et al. demonstrated that systemic injection of rGDF11 reverses age-related dysfunction in the skeletal muscle [5], more recent studies, published while our study was in progress, contradict this rejuvenating effect [15, 16]. In fact, the latter agree with previous publications describing GDF11 as a negative regulator of myogenesis [17, 18], similar to its homologue, myostatin. Two recent publications by Egerman et al. [15] and Rodgers and Eldridge [16] reported that some of the assays previously used to detect GDF11 [5] were not specific, and that GDF11 has an inhibitory effect on muscle regeneration inhibition and that levels of GDF11 actually increase with age. In response to this, Poggioli et al. have recently reported that the apparent age dependent increase in GDF11 levels, reported by Egerman et al. [15], is due to cross reactivity of the anti-GDF11 antibody with immunoglobulin, which is known to increase with age [19].

Despite this controversy in the activity of GDF11 in the context of aging, we addressed here whether GDF11 would have the ability to ameliorate the muscle wasting phenotype in the context of muscular dystrophy, by promoting muscle regeneration. Although daily doses of GDF11 treatment for 1 month resulted in elevated levels of this factor in the plasma, we were not able to detect any beneficial effect on the histology or strength in the muscles of treated dystrophic mice. We did not observe differences in terms of numbers of regenerating centrally nucleated myofibers. In contrast, we did observe an increase of collagen content, which denotes fibrosis, in the TA muscle of rGDF11-treated dystrophic mice compared to vehicle-injected controls.

## Conclusions

Our study shows no beneficial effect of treating dystrophic mice with rGDF11 and raises caution to a potential harmful effect, as shown by the pro-fibrotic outcome.

## Additional file

Additional file 1: Representative western blot analysis for rGDF11- and vehicle-injected *mdx* mice. Plasma was collected from *mdx* mice that had been treated with rGDF11 or vehicle for 30 days. (a) Equal amounts (75 μg) of protein for each mouse were loaded onto each lane. We used the GDF11 antibody from Abcam, which recognizes both the mature dimer (~25 kDa) and the monomer (~12.5 kDa) of both recombinant GDF11 and myostatin. We found increased levels of both the monomer and the dimer in the plasma of rGDF11-treated mice. Recombinant GDF11 (rGDF11) was loaded as positive control. (b) Stain-free detection of loaded proteins. (PDF 62 kb)

## Abbreviations

ANOVA: analysis of variance
BSA: bovine serum albumin
CSA: cross-sectional area
CTX: cardiotoxin
DMD: Duchenne muscular dystrophy
ECL: chemiluminescence
GDF11: growth differentiation factor 11
GDF8: growth differentiation factor 8
H&E: hematoxylin and eosin
IP: intraperitoneal
PFA: paraformaldehyde
PVDF: polyvinyl difluoride
rGDF11: recombinant GDF11
sFo: specific force
TA: tibialis anterior
TBS: Tris-buffered saline
TGF β: transforming growth factor β
