# Supplementary figures and images for: Treatment with rGDF11 does not improve the dystrophic muscle pathology of mdx mice
Source: Skelet Muscle. 2016 Jun 14;6:21. doi: 10.1186/s13395-016-0092-8 (PMC4906773; doi:10.1186/s13395-016-0092-8)

# Additional File 1

a

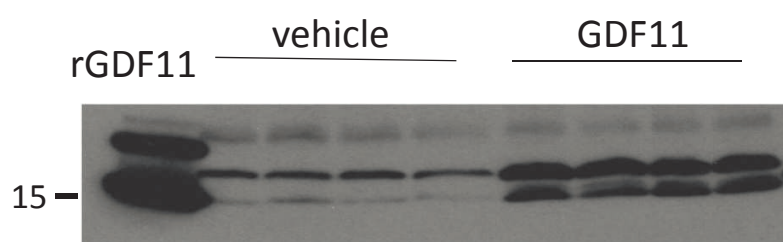

b

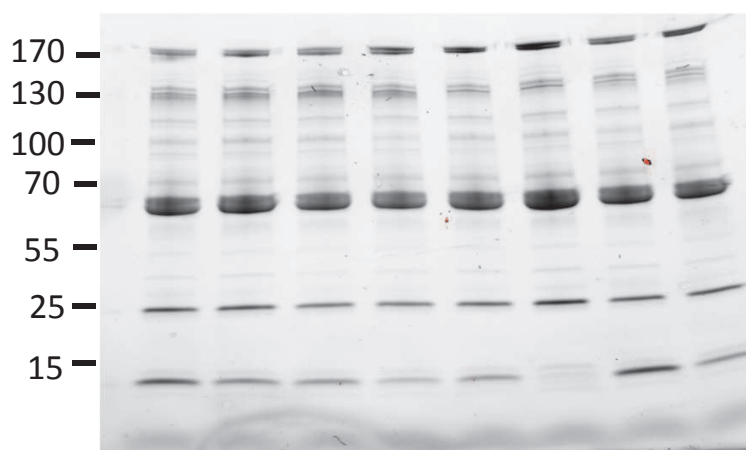

Supplement: Additional file 1: — Representative western blot analysis for rGDF11- and vehicle-injected mdx mice. Plasma was collected from mdx mice that had been treated with rGDF11 or vehicle for 30 days. (a) Equal amounts (75 μg) of protein for each mouse were loaded onto each lane. We used the GDF11 antibody from Abcam, which recognizes both the mature dimer (~25 kDa) and the monomer (~12.5 kDa) of both recombinant GDF11 and myostatin. We found increased levels of both the monomer and the dimer in the plasma of rGDF11-treated mice. Recombinant GDF11 (rGDF11) was loaded as positive control. (b) Stain-free detection of loaded proteins. (PDF 62 kb) [file 13395_2016_92_MOESM1_ESM.pdf]
